# Shane Godbolt, 1943–2019

**DOI:** 10.5195/jmla.2020.955

**Published:** 2020-07-01

**Authors:** Donna B. Flake

**Affiliations:** 1 donnabuntingflake@gmail.com, Wilmington, NC

## Abstract

Shane Godbolt, retired director of Partnerships in Health Information (PHI), died in her London home on November 24, 2019, surrounded by her family. Shane was a true partner to so many medical librarians to further the cause of international librarianship. Through her work in international librarianship, she made the world a better place.

(Laura) Shane Godbolt, retired director of Partnerships in Health Information (PHI), died in her London home on November 24, 2019, surrounded by her family. Shane was a dear friend and colleague, whose caring and support extended to medical librarians around the world.

(Laura) Shane Godbolt was born December 9, 1943, in Woking, England, United Kingdom, to Laura Kathleen and William Frank Spanner. Shane married Richard Godbolt in 1972, and they had two daughters, Miriam and Selina, and five grandchildren. She earned a bachelor of arts (BA) degree in history from Bedford College, University of London, in 1965. Her special area of interest was American history. In 1967, she received her postgraduate diploma in librarianship from the Polytechnic of North London.

Shane's first library position was at the medical library at St. Bartholomew's Hospital in London, where she worked for the remarkable medical librarian John Thornton, who greatly encouraged Shane in her new career.

In 1970, Shane became deputy librarian and subsequently director of the Charing Cross Hospital Medical School Library. It was at this time that Shane became interested in international librarianship, particularly in underserved parts of the world. The British Council sent her to India, where she saw the poverty with her own eyes. She met Indian medical librarians and could see their needs. Next, she made contacts in Sierra Leone and began the first of many trips to Africa to help medical librarians there. She sought funding and brought many African librarians to Britain for study tours.

**Figure:**
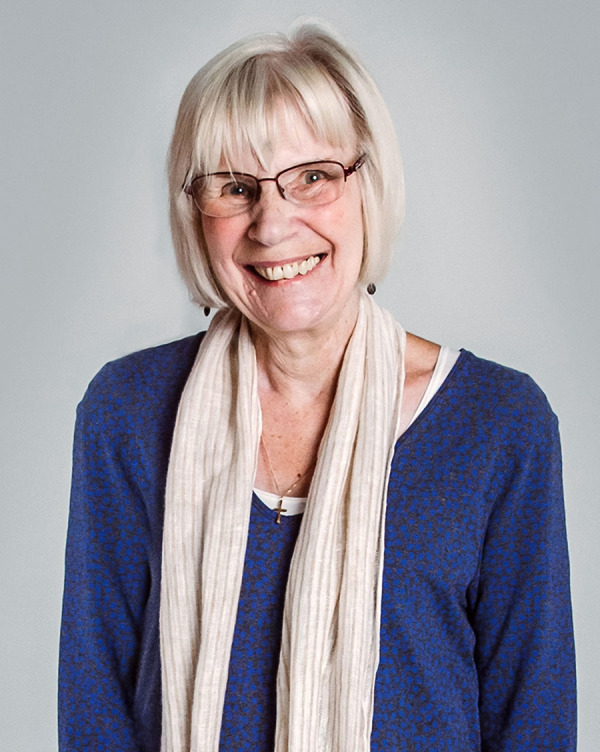


After leaving Charing Cross in 1992, she worked for the English National Health Service's North Thames Regional Library and Information Unit, latterly the London Library and Information Development Unit, as an advocate and spokesperson for medical libraries. Here Shane's knowledge, diplomacy, and poise were useful attributes.

Shane left the National Health Service in 2003 and turned her focus to what she loved the most: international health librarianship. In 1992, Shane became a founding trustee, chair from 2004 to 2006, and then director of the UK charity Partnerships in Health Information (PHI) from 2006 until her retirement in 2016. PHI focuses on the information needs of health care providers and the public, working directly with African organizations and networks to promote African leadership and support evidence-based practice. Shane did a great deal of work through PHI in Sub-Saharan Africa, helping medical librarians and health care providers in Ethiopia, Kenya, Nigeria, Sierra Leone, Tanzania, Uganda, and Zambia improve their access to reliable health information. Shane worked very closely with officers of the Pan-African organizations the Association for Health Information and Libraries in Africa (AHILA) and the Information Training & Outreach Centre for Africa (ITOCA).

British medical librarian Tom Roper described a typical day in Shane's life when she was director of PHI: “emails from Kenya, Sierra Leone and Ethiopia, two meetings at THET [the World Health Organization's (WHO's) Tropical Health and Education Trust], and a Skype conversation with the AHILA president calling from Nigeria.” During a weeklong visit with Shane in October 2015, I witnessed the intensity of her daily international activities alongside her added responsibility of coordinating a London study visit for two medical librarians from Nigeria.

Shane was a true partner to so many medical librarians to further the cause of international librarianship. For example, when I was organizing a delegation of medical librarians from Moldova to visit North Carolina for a study tour, she suggested that we could expand the trip and have the Moldovan librarians go to England to visit the Oxford Library and the Cochrane Collaboration. She contacted a few library vendors and got the support and funding needed to make this happen.

Shane was a very active member of the Medical Library Association (MLA) for many years, participating in the International Cooperation Section and greeting international librarians whenever possible. She was a founding member of the European Association for Health Information and Libraries (EAHIL); she was usually either a speaker or a continuing education course leader at EAHIL conferences. Former EAHIL President Suzanne Bakker commented that Shane often “took the opportunity to introduce a colleague from Africa in EAHIL meetings, and was always active in connecting people and promoting cooperation.” She continued: “Shane seemed indefatigable. From Shane I learned that medical librarianship is not just a job, but it is a way of life.”

As MLA's representative to EAHIL, I had countless conversations with Shane about the best ways to get EAHIL and MLA to communicate and collaborate. She continuously promoted MLA's involvement in librarianship internationally.

Shane was the creator, founder, and editor of the journal *Health Libraries Review (HLR),* now the *Health Information & Libraries Journal (HILJ).* Former *Bulletin of the Medical Library Association* Editor-in-Chief Susan Crawford, AHIP, FMLA, described *HLR*'s purpose: “to provide a forum for exchange of ideas and information in the field of medical librarianship and to publish original material reflecting current practice and new developments. *HLR* has become a successful highly-cited international journal.” A future special issue of *HILJ* will be dedicated to Shane's memory.

Over Shane's lifetime she has received numerous awards and honors. She was elected Associate of the UK Library Association (LA) in 1968; seven years later in 1975, she was awarded Fellowship of the LA. In 1986, she received the Cyril Bernard Memorial Prize for outstanding service to the profession. In 1998, the LA selected 100 individuals to receive their Centenary Award, and Shane was one of the recipients. Princess Anne, only daughter of Queen Elizabeth II, presented the awards, which read, “The LA Royal Charter Centenary 1898–1998.” She received an Honorary Membership in MLA in 2000, making her one of only three Britons to receive this honor. In 2020, she received the MLA T. Mark Hodges International Service Award posthumously.

Shane was very active in St. John the Baptist Anglican Church in Pinner, a suburb of London. She was a church warden for seven years and served regularly as a lay deacon at the Eucharist, only giving up her official duties in 2019. She continued to do *Bible* readings at Sunday services, until her health prevented her from doing so.

Shane had a tremendous dedication and great passion for international librarianship. She built an extensive network of contacts with her professional colleagues, both national and internationally. “She has left an important mark on international health information, and we are grateful that she has intersected with our lives,” Crawford said. “She is the quintessential achiever.”

Shane was a mentor, a colleague, an adviser, and a great inspiration to so many of us working in international librarianship. She had vast experience and knowledge, and set a very high standard in international librarianship. She had a warm and inviting personality and mesmerized every person she met. Everyone felt special in her presence. Shane's forte was building lasting bridges and friendships. She had the great capacity to inspire others with her ideas and to get others to help with international librarianship projects.

When medical librarians around the world heard of Shane's illness, they wanted to acknowledge her magnificent worldwide contributions. From Africa, she was awarded the 2019 AHILA Award. From Europe, she was awarded the EAHIL 2020 Award. Britain's Chartered Institute of Library and Information Professionals (CILIP) awarded her the 2019 Presidential Citation. The USA's MLA presented her a citation letter acknowledging her worldwide accomplishments in medical librarianship signed by MLA Executive Director Kevin Baliozian and MLA President Julia Esparza, AHIP. They characterized Shane as “a mentor and friend to countless MLA members, [who] has made lasting contributions to the association. It has been a privilege and an honor for MLA to work with Shane and to be able to recognize her countless endeavors.”

Just three months before Shane, I visited her, and we laughed, cried, and reminisced about all the great times. I casually mentioned that Irene (Rena) Machowa Lubker, AHIP, MLA's new representative to AHILA, lacked funding to attend the 2019 AHILA Conference in Africa. Immediately, Shane said, “Then I must help her.” She provided generous personal funding and called upon her friends and library vendors to contribute so that our MLA colleague was able to attend AHILA.

Former CILIP President Margaret Haines remembered this generosity of spirit as well. “Shane became my dear friend the day we first met,” she recalled. “She was the kind of colleague that always looked out for you and would do anything for you. Her generosity of time and spirit knew no bounds and it was contagious. She expected goodness and excellence from all of her colleagues.”

Although Shane never really retired from international librarianship, she did step back to spend more time with her family. Shane loved her old tabby cat Sebbie, and he spent much of his time in the final months of her life nestled beside her.

Through her work in international librarianship, Shane made the world a better place. Her tireless efforts helped so many librarians to learn and grow, and be excited about reaching out in the world.

